# The Quadripolar Left Ventricular Lead: An Effective Alternative for Phrenic Nerve Stimulation

**DOI:** 10.1016/s0972-6292(16)30604-0

**Published:** 2013-03-07

**Authors:** Krishnakumar Nair

**Affiliations:** Program Director, Cardiac Electrophysiology, University of Toronto

**Keywords:** phrenic nerve stimulation, quadripolar lead

Cardiac resynchronization therapy is no longer an exotic treatment modality for heart failure. With its role now being established even in mild heart failure, it is the standard of care either as bridging therapy for heart transplant or as destination therapy [1-4].

Despite the number of papers that suggest the benefit of optimization of location of left ventricular leads, every implanter knows the difficulty in going to an alternate lead position and location. Similarly, often, microdisplacement of the left ventricular (LV) lead post implant, can change grand success into abject failure forcing the electrophysiologist to sometimes actually turn off left ventricular pacing. Phrenic nerve stimulation (PNS) along the only posterolateral vein is not an uncommon situation where one has to back out from a procedure.

An LV lead with multiple electrodes thereby offering multiple pacing configurations is an obvious solution. Though there are a number of bipolar LV leads which offer upto three pacing configurations, the Quartet lead from St. Jude is perhaps the only 4 electrode LV lead which has 10 different pacing configurations available. Anecdotal experience with this lead has been heartening. Ohlow et al [5] in this issue of the journal systematize their experience with this lead. Not surprisingly, in their study, PNS never represented reason for failed LV pacing, neither acutely nor during follow-up. Successful biventricular pacing was achieved in as high as (24/26, 92%) cases.

Leads and devices with more bells and whistles come with a higher price tag. This may dissuade the operator from using this lead as the first line in every single case. A practical consideration would be the difficulty in passing all 4 electrodes into coronary sinus tributaries with acute angulations and multiple bends. A two electrode LV lead may have advantages in this situation. Another alternative in cases with PNS is to deploy an active fixation lead in a more proximal location. However, the commonly available active fixation lead is unipolar and not available in smaller diameters, and is suitable for deployment only in large sized tributaries. Epicardial LV lead placement is an option in severe heart failure, but would be high risk in post-operative hearts with sternal/ chest wall adhesions. The study though small with limited follow-up throws up some interesting questions. Would there be an advantage in increasing the number of electrodes further? Is there a point at which lead manueveribility becomes totally compromised? Only future research on these lines will give us the answers.
